# Singapore Statement on Global Health Security

**DOI:** 10.1136/bmjgh-2022-009949

**Published:** 2022-06-24

**Authors:** Adam Kamradt-Scott, Yik Ying Teo, Rebecca Katz

**Affiliations:** 1 School of Transnational Governance, European University Institute, Firenze, Toscana, Italy; 2 Saw Swee Hock School of Public Health, National University Singapore, Singapore; 3 Center for Global Health Science and Security, Georgetown University, Washington, DC, USA

**Keywords:** Health systems, Health policy

Global Health Security (GHS) is a state of freedom from the scourge of infectious diseases irrespective of their origin or source. As the COVID-19 pandemic has shown, however, it can only be achieved and maintained through concerted, cooperative action at all levels—from local communities to the international system—when acting in solidarity, informed by equity.

We reaffirm the Sydney Statement on GHS’s seven core principles (The seven principles are: (1) That interventions must be inclusive, equitable and data driven; (2) Prevention, detection and response capabilities are critical and must be linked with universal health coverage and health system strengthening; (3) Governments must comply with the International Health Regulations and other associated agreements; (4) GHS requires action and engagement from all sectors; (5) GHS must embrace a One Health approach; (6) Countries with resources have a duty to partner with those with lower capacity to strengthen capabilities; and (7) Sustainable, comprehensive funding is critical.). These principles, agreed prior to the pandemic, are only more salient now. COVID-19 has highlighted the urgent need for strengthening and maintaining global, regional, national, and local preparedness and response capacities, even as we also must prepare for known and hitherto unforeseen challenges emerging from a changing climate, microbially resistant microorganisms, existing pathogens and future zoonotic spillover events. We must embrace the fact we live together on one planet, deeply connected to each other and our natural environment.

To that end, we declare:

1. GHS is made sustainable only when embedded within universal health systems that ensure equitable access to services and treatment, irrespective of socioeconomic status or means to pay, and delivered by an appropriately trained and remunerated health workforce.
2. Health emergency planning, preparedness and response workforces must be strengthened and proactively expanded to include multisectoral, interdisciplinary expertise. To foster these workforces, inclusive, lifelong training must be encouraged, readily accessible and linked to professional advancement.
3. Preventing future unintended disease events requires enhanced surveillance of animal and environmental conditions, including in urban settings, and the adoption of policies and standards that proscribe activities that harm the natural environment.
4. Governments must sustainably finance, build, strengthen, maintain and regularly practice preparedness and response capabilities that respect human rights, are inclusive, context specific, and adhere to relevant international law such as the International Health Regulations.
5. Preparedness and response must strengthen and maintain not only technical but also social, economic and political measures for reducing harm arising from disease events. This includes taking practical steps to improve health literacy across communities to mitigate misinformation and disinformation, and the development of new social, economic and political preparedness indicators to enhance resilience.

Achieving GHS, strengthening health systems and improving population health around the world requires a sustained commitment at all levels of government and across all sectors of society.

## Data Availability

Data are available in a public, open access repository.

